# Taxonomic and Functional Diversity of Benthic Macroinvertebrate Assemblages in Reservoirs of South Korea

**DOI:** 10.3390/ijerph20010673

**Published:** 2022-12-30

**Authors:** Da-Yeong Lee, Dae-Seong Lee, Young-Seuk Park

**Affiliations:** Department of Biology, College of Science, Kyung Hee University, Dongdaemun, Seoul 02447, Republic of Korea

**Keywords:** macroinvertebrate assemblage, community metric, taxonomic, functional diversity, community index, community diversity index, biological monitoring, self-organizing map

## Abstract

Numerous community indices have been developed to quantify the various aspects of communities. However, indices including functional aspects have been less focused on. Here, we examined how community composition varies in response to the environment and discovered the relationship between taxonomic diversity and functional diversity while considering the environment. Macroinvertebrate communities were collected from 20 reservoirs in South Korea. To characterize functional diversity, functional traits in four categories were considered: generation per year, adult lifespan, adult size, and functional feeding groups. Based on their community composition, we classified the reservoirs using hierarchical cluster analysis. Physicochemical and land use variables varied considerably between clusters. Non-metric multidimensional scaling indicated differences between reservoirs and clusters in terms of structure, functional diversity, and environmental variables. A self-organizing map was used to categorize functional traits, and network association analysis was used to unravel relationships between functional traits. Our results support the characteristics of species’ survival strategies such as r- and K-selection. Functional richness exhibited a relationship with taxonomic diversity. Our findings suggest that different types of diversity could play complementary roles in identifying biodiversity. Our findings should prove useful in developing new criteria for assessing freshwater ecosystem health, as well as in evaluating and predicting future alteration of benthic macroinvertebrate communities facing anthropogenic disturbances.

## 1. Introduction

To quantify the features of communities and ecosystems, various community indices have been developed. Most indices have traditionally been based on taxonomic richness and abundance, giving rise to taxonomic diversity indices such as the Simpson diversity index [1] and the Shannon diversity index [2]. Taxonomic diversity has the advantage of providing numerical values immediately, without the need for any additional measures other than identification [3]. Based on this advantage, taxonomic diversity has been widely used in various types of biological indices to evaluate ecosystem responses to environmental changes and disturbances [4]. Although taxonomic diversity properly depicts the condition of the systems under consideration, it does not present ecosystem functioning, which is one of two key aspects (i.e., structure and functioning) in ecosystems.

The life history of species reflects their adapted traits in both biological and ecological aspects through natural selection to their environment [5]. Therefore, the functional traits of species display the characteristics of responses of organisms to their environment and are key for understanding community characteristics that are difficult to determine using taxonomic community indices alone [5]. Functional diversity is defined as the diversity of functional traits in a community, and it comprises components of biodiversity that impact how an ecosystem operates or functions [6]. Mason et al. [7] proposed three popular functional diversity indices, namely functional richness, functional evenness, and functional divergence; Villéger et al. [8] developed these concepts further. 

Functional traits have been proposed as biomonitoring tools because they reflect environmental conditions [9]. Accordingly, a variety of functional approaches based on functional traits have been used to evaluate ecosystem assessments in various ecosystems with different types of organisms (e.g., plants, plankton, macroinvertebrates, and fish) [10], including functional feeding groups in macroinvertebrates [11]. Functional characteristics and niche show how creatures that have adapted to their environment are connected to functional communities, and functional traits show how an organism’s life history has been shaped by the environment [12,13]. 

Tomanova et al. [14] employed the functional traits of macroinvertebrates to evaluate biotic integrity. Using both taxonomic and functional diversity, Villéger et al. [15] demonstrated that various trophic fish communities differently respond to habitat alteration. Taxonomic and functional diversity describe various ecosystem features; thus, functional diversity, in addition to taxonomic diversity, could be employed as an assessment criterion for ecosystem functioning and biodiversity [16,17]. Different functional properties can reveal various elements of an ecosystem. 

Benthic macroinvertebrates are sedentary, live in a variety of environmental conditions, and play a vital role in the food trophic structure of aquatic communities by linking producers, apex predators, and decomposers [18]. They have an intermediate lifespan, spanning from months to a few years, and a wide range of functional trait dimensions [9,19]. Therefore, macroinvertebrates can reflect environmental conditions in aquatic ecosystems and have been widely used in aquatic ecosystem assessments [20,21]. The responses of functional traits and diversity of benthic macroinvertebrates to environmental gradient can present functional properties of ecosystems. Understanding the relationship between taxonomic diversity and functional diversity along an environment gradient is important because anthropogenic disturbance is increasing and our ecosystem management focuses on disturbed habitats [22]. 

Many studies were conducted on taxonomic diversity and functional diversity responding to different environmental conditions, but there are limited studies on benthic macroinvertebrates in reservoirs [23]. Recently, Coccia et al. [24] proposed the functional diversity of macroinvertebrates as a tool to evaluate wetland restoration. In particular, there is no research on functional approaches with macroinvertebrates in reservoirs in South Korea. Therefore, we conducted this study to test two hypotheses: Taxonomic diversity and functional diversity have a strong relationship and functional traits and diversity are influenced by the environmental condition in reservoirs. Specifically, we examined the differences of community composition along an environmental gradient, the relationship between functional traits related with the environment, and the relationship between taxonomic diversity and functional diversity along an environmental gradient in reservoirs. 

## 2. Materials and Methods

### 2.1. Ecological Data

**Figure 1:**
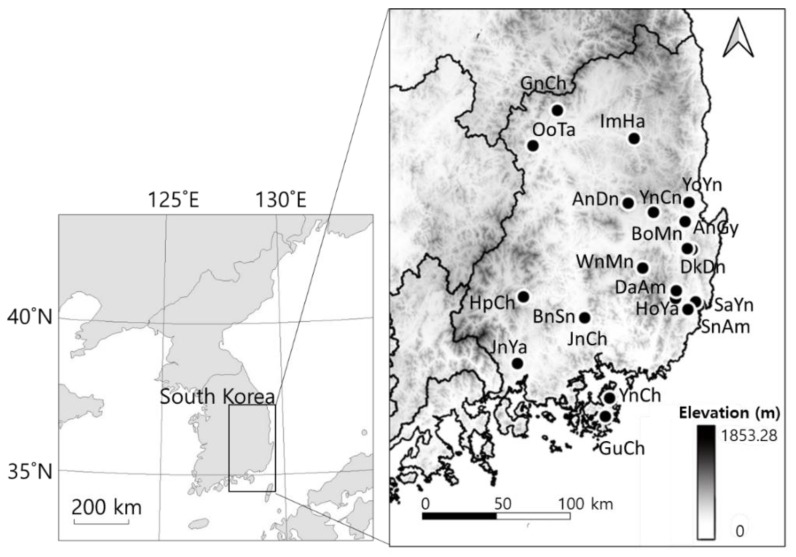
Location of study reservoirs in South Korea.

A dataset of benthic macroinvertebrates, which were surveyed at 20 reservoirs in the Nakdong River catchment, South Korea (Figure 1, Table S1) from 2009 to 2017, was obtained from reports of the Survey on the Environment and Ecosystem of Lakes (SEEL) [25]. The Nakdong River flows into the southern part of the Korean Peninsula and is the longest river in South Korea (total river length: 8567 km), with a catchment area of 30,047 km^2^ [26,27]. Macroinvertebrates were collected twice a year (in spring and autumn) using a dredge net (40 cm width, 0.5 mm mesh size) [25]. According to the SEEL protocol [25], samples were collected at two sampling points where water was present throughout the year in each reservoir [28], and the samples were pooled for each reservoir, and average abundance (individuals m^2^/sample/year) data were used in the analyses. The final dataset consisted of 90 benthic macroinvertebrate taxa from 20 reservoirs.

To evaluate differences in communities based on environmental differences, 19 environmental variables in three categories were used: 12 water quality variables (pH, dissolved oxygen (DO), biochemical oxygen demand (BOD), chemical oxygen demand (COD), total suspended solids (TSS), total nitrogen (TN), total phosphorus (TP), total organic carbon (TOC), water temperature (WT), electric conductivity (EC), chlorophyll-a (Chl-a), and transparency), four geomorphological variables (elevation, storage area, bank height, and circumference) and three land use types (proportions of urban area, agricultural area, and forest). Water quality data were obtained from the Water Environment Information System (WEIS; http://water.nier.go.kr/, accessed on 7 June 2021) of South Korea of the Ministry of Environment in South Korea. Water quality was measured every month at each reservoir according to the WEIS protocol. We used average values of water quality variables in the sampled year in the analyses. Land use categories within a catchment of the study reservoirs were measured from a digital map using QGIS [29]. Physical environmental data were obtained from the Water Management Information System (WAMIS; http://www.wamis.go.kr/, accessed on 16 November 2022).

### 2.2. Functional Traits of Macroinvertebrates

Based on the literature, each taxon of macroinvertebrates was characterized by functional traits in four categories, including the number of generations per year (voltinism), adult lifespan, adult size to maturity, and functional feeding groups of taxa at the genus level (Table 1) [30,31,32,33,34,35]. References used to define functional traits were given in the supplementary information Table S3. The number of generations per year, adult life span, and adult size are related to the population dynamics of the species, whereas functional feeding groups are related to food availability. Voltinism aided in determining the diversity of the age structure, which influences the response patterns of the population to disturbances [36]. Adult life span is related to the reproductive potential of a species [37]. In contrast, adult size incorporates ecological and physiological responses to the local environment and is related to fecundity [38].

### 2.3. Data Analyses

#### 2.3.1. Taxonomic Diversity and Functional Diversity

Taxonomic community indices such as taxa richness, abundance, the Shannon diversity index [2], evenness, and the dominance index were calculated for each study reservoir. In addition, functional traits were used to calculate functional diversity such as functional richness (FRic) and functional evenness (FEve) [8,9]. FRic is calculated using the convex hull volume and represents the potential occupancy of niche space [7,8], and FEve is based on the minimum spanning tree and indicates how average species traits are distributed regularly within the occupied trait space [8].

#### 2.3.2. Community Characteristics

To investigate how well the community structure characterizes the reservoirs, a two-way cluster analysis [39] based on community composition was conducted using the Ward linkage method and Bray–Curtis distance. To reduce variation, abundance was log-transformed, and only taxa found at more than two reservoirs (52 taxa) were used in the analyses. One-way analysis of variance (ANOVA) was used to compare differences in environmental conditions among clusters, followed by a post hoc Tukey’s HSD multiple comparison test [40]. Non-metric multidimensional scaling (NMDS) was performed using the same data employed in the cluster analysis to determine the relationship between spatial variation in community structure, taxonomic diversity indices, functional diversity indices, and environmental variables [41].

#### 2.3.3. Functional Characteristics of Communities

The communities were analyzed based on the functional traits of species, and the relationships between functional and taxonomic diversity were compared. We used functional traits in four categories (number of generations per year, adult lifespan, adult size, and functional feeding groups), and the analysis included 85 taxa with information of at least three functional trait categories.

To determine the associations between taxa and functional traits, a self-organizing map (SOM) [42] was constructed using 85 taxa data comprising functional traits. The functional traits of each taxon were expressed as a dichotomous transformation (i.e., 0 or 1). SOM output units (40 = 5 × 8) were calculated based on the function $5\times \sqrt{\left(\mathrm{number}\;\mathrm{of}\;\mathrm{samples}\right)}$ [43,44]. Following the learning process of the SOM, taxa and functional traits were categorized using two-way cluster analysis with the Ward linkage method and the Euclidean distance measure [45]. 

A network association analysis was performed to evaluate the links between functional attributes. Network association indices such as support, confidence, and lift were used to characterize the association among functional traits. Support for item X was defined as the ratio of transactions in the dataset that contained the item [46]. If items X and Y were present, support (X→Y) refers to the proportions that contained both items X and Y (P (X ∩ Y)). Confidence (X→Y) denotes the conditional probability that Y will be adopted if X is adopted (P (X ∩ Y)/P(X)) [46]. The maximum confidence level was 1, indicating that Y was always adopted when X was adopted. Lift (X→Y) is the ratio of the probability of choosing Y when choosing X to the probability of choosing Y (P (X ∩ Y)/P(X) P(Y)) [47]. This indicates the strength of the link between X and Y. Rules with one-on-one correspondence between functional traits were used with 0.1 of minimum support and 0.8 of minimum confidence as default as well as 0.05 of minimum support and 0.5 of minimum confidence to include more associated networks.

The Spearman rank correlation coefficient was calculated to evaluate the relationship between taxonomic and functional diversity indices. Furthermore, a generalized additive model (GAM) was employed to characterize changes in taxonomic and functional diversity as a function of community indices by accounting for the gradient of environmental variables [48].

All statistical analyses were conducted using the R software (https://www.r-project.org/, accessed on 23 June 2022) with the following related packages: functional diversity indices with the dbFD function in the FD package [49,50], hierarchical cluster analysis and NMDS with the vegan package [51], SOM with the kohonen package [52], ANOVA with the stat package [53], Tukey’s HSD multiple comparison test with the PMCMRplus package [54], network association analysis with the arules package [55], and GAM with the mgcv package [56,57].

## 3. Results

### 3.1. Patterns of Taxonomic Diversity

*Chironomus* spp. was the most abundant taxon in the dataset (62.9% of total abundance, 10,174 inds/m^2^) at the 20 sites, followed by *Limnodrilus* spp. (1344 inds/m^2^ at 20 sates) and *Palaemon paucidens* (867 inds/m^2^ at 8 sites). *Macrobrachium nipponense* (15 sites) and *Ecnomus tenellus* (12 sites) all had high occurrence rates. 

**Figure 2:**
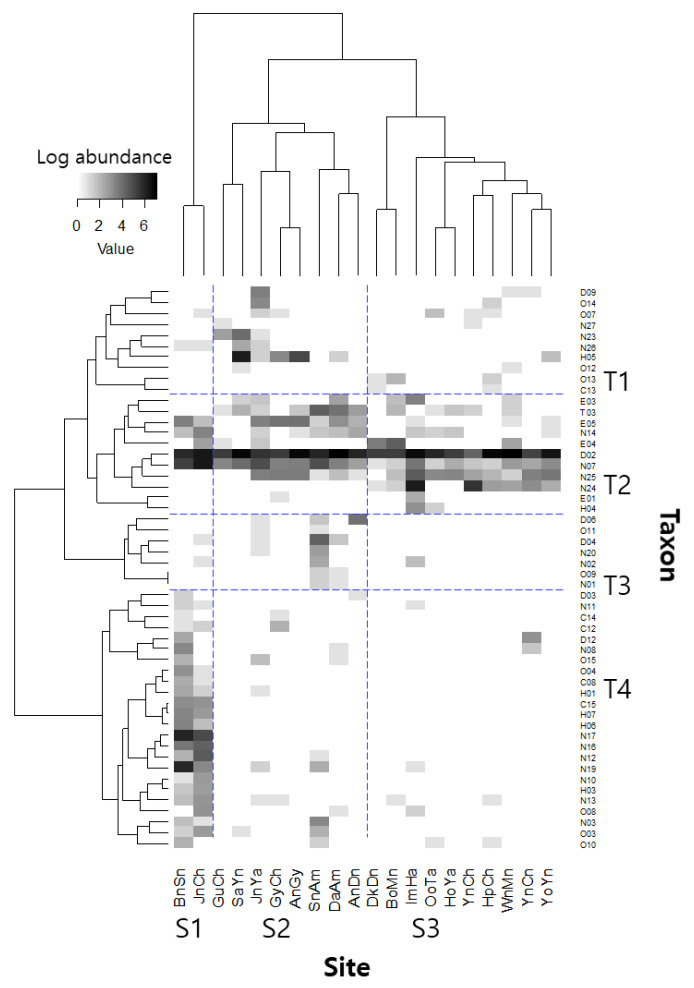
Two-way cluster analysis of the study sites based on similarities of community composition using Ward’s linkage method with the Bray–Curtis distance measure. Letters and numbers placed vertically denote the individual taxa presented in the Supplementary Information Table S2.

Based on the similarity of their community compositions, the 20 reservoirs were divided into three clusters (S1–S3; Figure 2). Furthermore, the 52 taxa identified in the dataset were classified into four groups (T1–T4) based on similarities in their abundance and occurrence in the study reservoirs. The dominant taxon in all clusters was *Chironomus* spp.; however, the second dominant taxa varied by cluster, such as *Limnodrilus* spp., *Micronecta* spp., and *P*. *paucidens* in clusters S1 through S3, respectively. In group T4, cluster S1 (BnSn and JnCh) had greater taxonomic richness and abundance. The taxa in group T2 were widely distributed across the study reservoirs. Taxonomic abundance was low in T1 and T3. 

**Figure 3:**
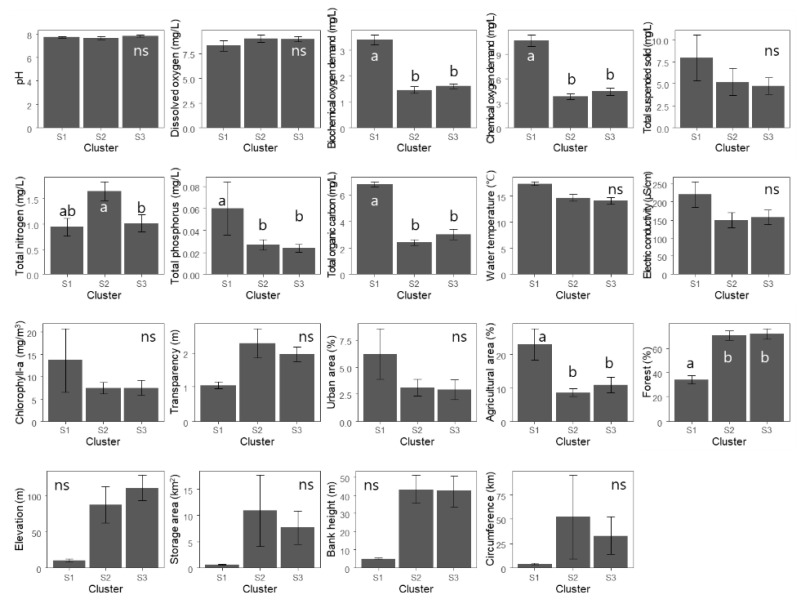
Differences between environmental variables between clusters classified in Figure 2. Different letters denote statistical differences based on Tukey’s HSD test (*p* < 0.05). Error bars indicate standard errors. ‘ns’ means no significantly different among clusters.

Physicochemical environmental conditions varied across the reservoirs (Figure 3). Among water quality variables, BOD, COD, TP and TOC were the highest in cluster S1 (ANOVA, *p* < 0.05), whereas TN was the highest in cluster S2 (ANOVA, *p* < 0.05). Regarding land use, the proportion of forest was the lowest and that of the agricultural area was the highest in cluster S1 (ANOVA, *p* < 0.05). Cluster S1 had higher EC and lower transparency, elevations and bank heights than the other clusters, although these differences were not significant (ANOVA, *p* = 0.14 for both elevations and bank heights).

**Figure 4:**
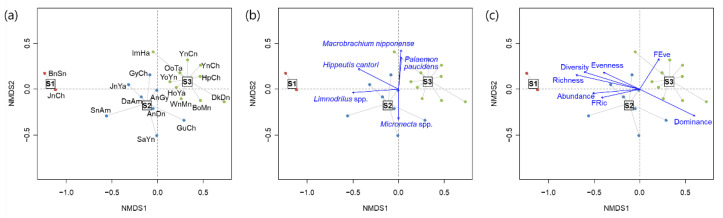
Non-metric multidimensional scaling (NMDS) ordination for the study sites based on taxonomic abundance (stress value: 0.1168) (**a**), biplots with dominant species (**b**), and community indices (**c**). Richness: taxa richness, Evenness: taxa evenness, Dominance: taxa dominance, Diversity: Shannon diversity index, FRic: functional richness, and FEve: functional evenness.

**Figure 5:**
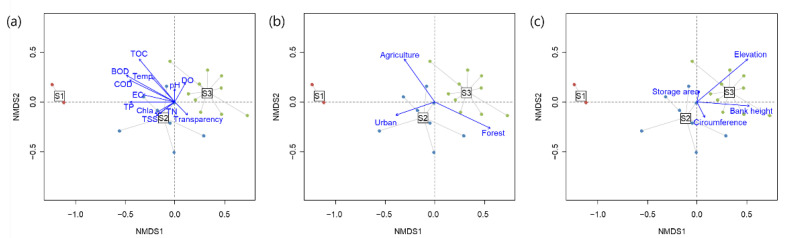
Biplots with 19 environmental variables in three categories on the NMDS ordination in Figure 4. (**a**) Physicochemical water quality variables, (**b**) land use types, and (**c**) geomorphological variables. DO: dissolved oxygen, BOD: biochemical oxygen demand, COD: chemical oxygen demand, TSS: total suspended solid, TN: total nitrogen, TP: total phosphorus, TOC: total organic carbon, WT: water temperature, EC: electric conductivity, Chla: chlorophyll-a, Urban: proportion of urban area, Agriculture: proportion of agricultural area, and Forest: proportion of forest landscape.

The NMDS ordination reflected differences in the communities and the environmental conditions of the reservoirs (Figure 4 and Figure 5). Cluster S1, which had a distinct community composition, was isolated from the other clusters (Figure 4a). The second and the third most dominant taxa in each cluster characterized the differences in the clusters (Figure 4b). Axis 1 of the NMDS was positively correlated with dominance and FEve, and negatively correlated with richness, abundance, Shannon diversity, and FRic (Figure 4c). However, FEve and dominance had positive and negative correlations along NMDS axis 2, respectively. Nutrient conditions (BOD, COD, TP and TOC) and the proportion of agricultural area were higher in cluster S1 than in the other clusters, whereas the proportion of forest and elevation were lower in cluster S1 (Figure 5). Moreover, elevation and the proportion of forest were high in clusters S2 and S3. 

### 3.2. Functional Traits and Diversity

**Figure 6:**
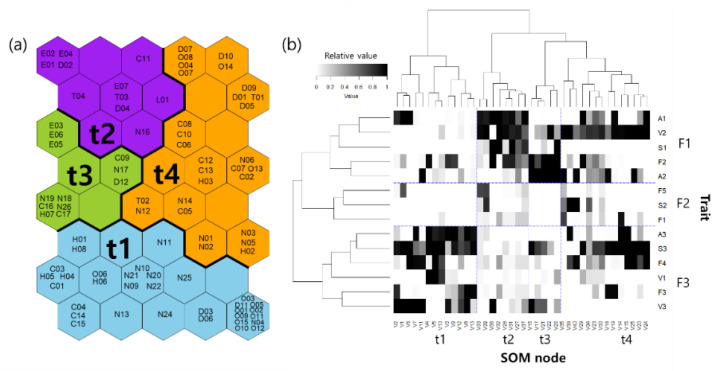
(**a**) Classification of taxa using self-organizing map (SOM) algorithms based on functional traits. Letters and numbers denote the individual taxa presented in the supplementary information Table S2. (**b**) Two-way classification of functional traits (vertical arrangement; groups F1–F3) and SOM units of traits (horizontal arrangement; clusters t1–t4) via hierarchical cluster analysis utilizing the Ward linkage method using Euclidean distance of classification. Weight vectors of SOM output units were used for the two-way cluster analysis. Abbreviations of traits are defined in Table 1.

The 85 taxa were classified into four groups (t1–t4) through the SOM learning process based on similarities in their functional trait composition (Figure 6). More than half of Hemiptera taxa were in group t1. Non-Insecta taxa, Diptera, Coleoptera and Odonata were typically included in groups t1 and t4, whereas Ephemeroptera taxa were in groups t2 and t3.

Based on the weight vectors of the trained SOM, functional traits were classified into three clusters (F1–F3; Figure 6b). In F1, taxa in groups t2 to t4 exhibited a highly frequent occurrence of functional traits similar to those in V2; in F2, infrequent functional traits were included similar to those in F1, F5 and S2; in F3, taxa in group t1 and t4 had a high frequency of functional traits such as S3.

Group t1 was characterized by large body sizes (S3; Figure 6b), and the functional feeding groups differed between taxa. Group t2 consisted of taxa with small body sizes (S1) and short lifespans (A1), as well as one generation per year (V2). Group t3 was composed of gatherer-collectors (F2) with one or more generations (V2–V3) per year and medium lifespans (A2). Patterns in group t4 with one generation per year (V2) were distinguished by large body sizes (S3), as well as various feeding habits.

**Figure 7:**
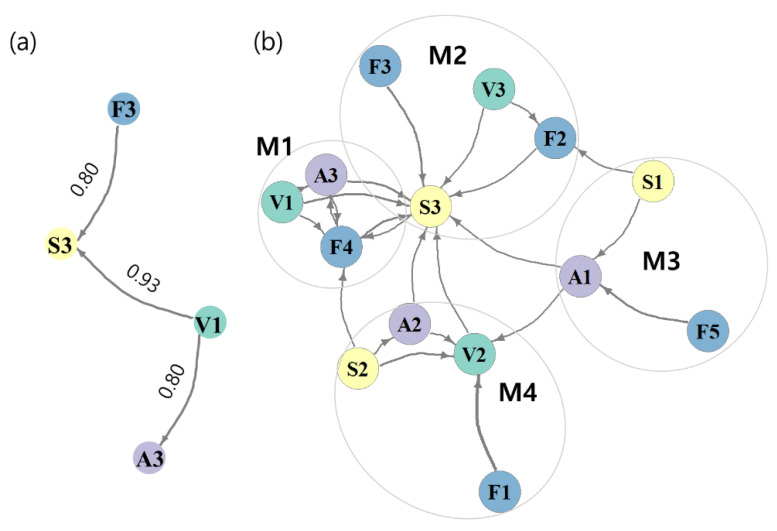
Results of the co-occurrence network analysis based on the association between traits. Link width is proportional to confidence. (**a**) Minimum support was 0.1, and minimum coefficient was 0.8. Numbers adjacent to links indicate confidence. (**b**) Minimum support was 0.05, and minimum confidence was 0.5. The circles denote modules of the traits (modularity: 0.32). The nodes indicate abbreviations of the traits listed in Table 1.

Network association analysis revealed relationships between functional traits (Table 2, Figure 7). Three distinct associations were identified with minimum support of 0.1 and minimum confidence of 0.8. Herbivorous feeding pattern (F3) resulted in a large body size (S3). Less than one generation per year (V1) also associated with large body size (S3) and related to long lifespans (A3; Figure 7a). The network association results supported those of the hierarchical cluster analysis (Figure 6b), especially functional traits in group F3. Moreover, four modules with modularity of 0.32 were generated with minimum support of 0.05 and minimum confidence of 0.5 (Figure 7b). Module M1 showed patterns similar to those of the functional trait cluster analysis in group F3 (Figure 6b). Taxa with fewer than one generation per year (V1) were associated with predatory feeding patterns (F4) and long lifespans (A3). Herbivores (F3) had a large body size (S3), whereas small body size (S1) resulted in gatherer-collectors (F2) and short lifespans (A1). The characteristics of more than one generation per year (V3) and gatherer-collector feeding habits (F2) were linked. Moderate characteristics, shredders (F5), and filterer-gatherers (F1) were included in modules M3 and M4, respectively. Members of module M4 were moderate characteristics.

### 3.3. Relationship between Diversity Indices

**Figure 8:**
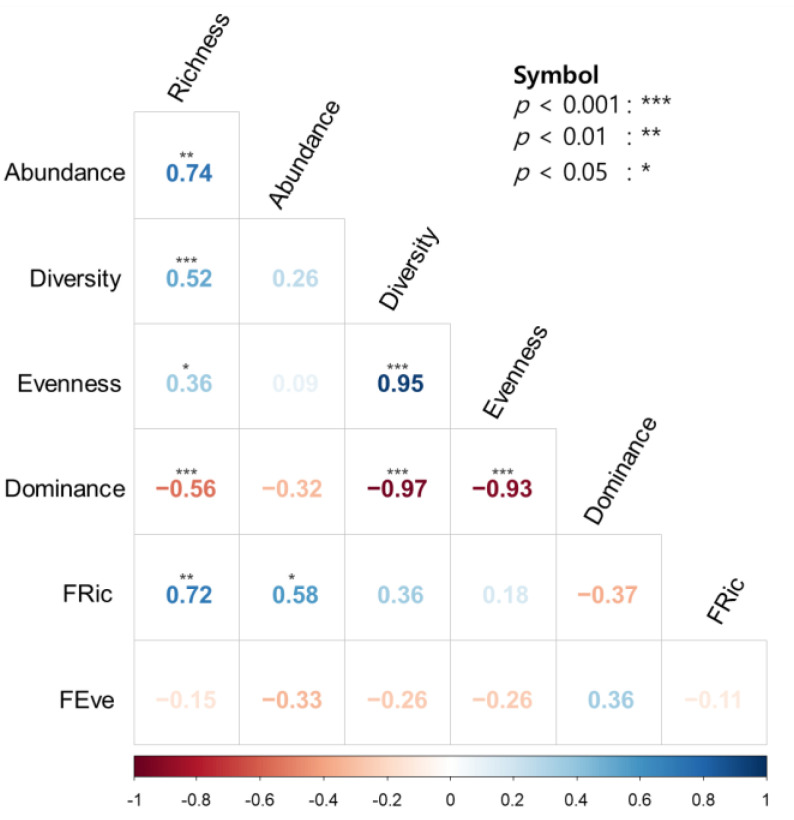
Spearman rank correlation between taxonomic and functional diversity indices. Acronyms are given in Figure 4.

There was a positive correlation between richness and abundance (*r* = 0.74, *p* < 0.01), between richness and the Shannon diversity index (*r* = 0.52, *p* < 0.01), between richness and evenness (*r* = 0.36, *p* < 0.05), and between evenness and the Shannon diversity index (*r* = 0.95, *p* < 0.01) among the taxonomic diversity indices (Figure 8). Dominance was negatively correlated with richness, the Shannon diversity index, and evenness (*r* = −0.97–−0.56, *p* < 0.01). The functional community indices FRic and FEve were not significantly correlated with each other (*r* = −0.11, *p* = 0.62). Moreover, the functional diversity index FRic was positively correlated with richness (*r* = 0.72, *p* < 0.01) and abundance (*r* = 0.58, *p* < 0.05). However, FEve was not significantly correlated with taxonomic community indices (*r* = −0.33–0.36, *p* > 0.18), although it was weekly correlated with dominance (*r* = 0.36, *p* = 0.43).

**Figure 9:**
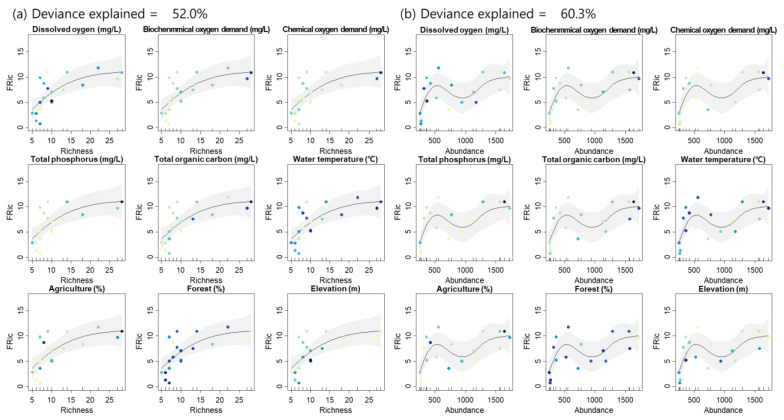
Relationship between taxonomic (taxa richness (**a**), abundance (**b**)) and functional diversity and environmental variables. Only statistically significant relationships are plotted. GAM was applied to analyze the relationship between taxonomic and functional diversity (black lines) with 95% confidence intervals (grey-shaded areas). Point color presents relative value of each environmental variable (light: low value and dark: high value).

With the indices demonstrating significant correlations (Figure 8), the relationships between taxonomic and functional diversity indices were analyzed further by taking environmental factors into account (Figure 9). Richness and abundance explained 52.0% and 60.3% of the deviance in FRic by GAM, respectively. Environmental variables tended to increase or decrease as taxa richness, abundance and FRic increased. Taxa richness, abundance and FRic were positively correlated with BOD. TP was positively related to taxa richness and FRic, whereas these indices negatively correlated with proportion of forest and elevation.

## 4. Discussion

We evaluated the association between the taxonomic and functional diversity of benthic macroinvertebrates with environmental variables in 20 reservoirs. By linking environmental conditions, we were able to identify changes in community structure in response to environmental changes. Linkages between functional features and the association between biodiversity indicators were also identified.

### 4.1. Taxonomic Community Structure

Water quality has a significant influence on the composition of taxonomic communities. In particular, groups were separated by the parameters BOD, COD, TN, TP and TOC (Figure 3). Based on the water quality standard of South Korea (https:/water.nier.go.kr/, accessed on 7 June 2021), differences in COD, TN, TP and TOC standards existed among the study reservoirs. In this study, the BnSn and JnCh reservoirs (S1) had a distinct community composition as compared to the other reservoirs (Figure 2). Cluster S1 (BnSn and JnCh) also had a relatively higher BOD, with an average of 3 mg/L as compared to the other clusters (whose BOD was approximately 2 mg/L), highlighting the effects of BOD on community composition (Figure 3). 

There were numerical disparities in EC and transparency across clusters (Figure 3), which influenced community structure in various ways. EC indicates the overall water quality by integrating the total number of dissolved ions in water. On the other hand, the transparency of water influences food web dynamics in aquatic ecosystems [58] and functional traits communities, such as foraging strategies and success [59]. 

TN, TP and TOC are known to be closely related to community composition [60]. Nitrogen and phosphorus enable tolerant species to thrive in lakes [61]; in particular, TP regulates the taxonomical and functional beta diversity of macroinvertebrates [60]. In addition, TOC is influenced by inputs from the watershed [62]. Water quality is closely related to land use and cover, in which the proportions of the urban landscape, agricultural land, and forest areas are particularly important [63,64]. In our study, we found that the landscape had a distinct composition in S1, where the community differed from those in the other clusters (Figure 3). In addition, landscape composition showed similar or opposite tendencies to water quality variables such as BOD, COD, and TOC (Figure 5).

The ecosystem health index based on benthic macroinvertebrates is closely related to chemical and physical gradients and displays a strong correlation with land use, suggesting that land use indirectly induces considerable changes in macroinvertebrate communities [65]. In inland wetlands, the main drivers of water quality degradation are nutrient and sediment inputs, which are associated with altered land use [66]. Human-induced land use has been the major source of nitrogen and phosphorus in lakes, while forests served to reduce nitrogen and phosphorus contents in lakes [67].

### 4.2. Relationships between Functional Traits of Macroinvertebrates 

Functional traits were classified by the SOM learning process based on the presence or absence of traits in the observed species, and their association was analyzed using network association analysis (Figure 6b and Figure 7). The taxonomic system reflects biological characteristics in the community [32], whereas the functional traits of species, such as the combination of size, reproductive traits (r to K strategies), and food and feeding habitats, could explain taxonomic groups because functional traits reflect the evolutionary responses of species to their environmental changes [68]. Some functional traits have similarities or trade-offs, such as body size and fecundity [38]. 

Even though the taxonomic system and biological characteristics are weakly related [32,68], the classification of functional traits was not completely consistent with that of the taxonomic characteristics (Figure 6), indicating that functional and taxonomic community functions differ. Taxa that survive environmental filtering have several linked traits that allow them to adapt well to their environment [69]. Our findings were consistent with the characteristics of r- and K-selection taxa [70]. Rapid development and early reproduction, and short lifespan are related to r-selection, whereas K-selection taxa are related to slower development, delayed reproduction, large body size, and long lifespan. High voltinism is caused by a combination of fast development and rapid reproduction.

There is a trade-off between body size and voltinism [71], which is reflected in the findings of our study (Figure 6b and Figure 7). Although Zeuss et al. [71] found that climate could influence this relationship, our study sites had a comparable climate; therefore, a minor temperature variation may not have affected this relationship in this study. The close size-voltinism relationship is usually valid in areas with similar climates. Species with frequent voltinism grow quickly, and the energy necessary for rapid growth lowers the priority of other abilities [72].

Body size and trophic level are closely linked in aquatic ecosystems [73]. In this study, large body size was closely related to predatory feeding habits, whereas small body size was related to gatherer-collector feeding habits (Figure 7). Gatherer-collectors, which feed on organic matter or primary producers, have become the primary food source for predatory insects [74], and have a low trophic level. Large species require more energy than small species since they require more energy to grow. Predators tend to have a larger body size and require a high-caloric energy source (food). As the size of the predator increases, prey size tends to increase [75]; the consumption rate also increases with the predator body mass [76]. Therefore, predator size appears to be directly related to food consumption. 

Taxa with high trophic levels and predatory feeding habits are density-dependent on prey in that food energy efficiency is high, but quantity is limited [77]. Density dependence is observed not only in interspecific competition but also in intraspecific competition. There are limitations, however, because size-dependent intraspecific predation occurs between individual predators with low voltinism; therefore, larvae have varied size structures [78]. Furthermore, the larger the body size, the smaller the intraspecific adult size variation, implying that a large-sized species requires a high-quality habitat in which body size may grow; hence, the suitability of the environment could be evaluated by the presence of large predators [79].

Movement capability is related to tolerance to environmental changes [80], and tolerance is associated with life span. A taxon with a reduced lifespan is vulnerable to environmental changes [80], seemingly due to difficulties in surviving for a sufficient period of time until a suitable environment is built; therefore, they are replaced by species with greater fitness. In consideration of the characteristics of r-selection species with short life spans, r-selection species with a higher number of voltinisms are hypertrophied in a fit environment, or do not fit and occur less frequently. Therefore, in the absence of major disturbances, a community with longer-lived species shows less variation in community structure and decreases the temporal heterogeneity of the community [81]. Considering the slow growth and long lifespans of K-selection species, their presence at moderate perturbation aids in lowering community heterogeneity, since slow-growing species are more readily adapted to disturbances [82]. As such, functional traits also influence temporal changes in community structure.

### 4.3. Taxonomic Diversity and Functional Diversity

Among the correlations between taxonomic and functional diversity indices, FRic was related to taxonomic indices, whereas FEve was not (Figure 8). Taxonomic and functional diversity have a positive relationship, although the two are partially independent [83]. Although Hacala et al. [84] showed that taxonomic and functional diversity were strongly positively correlated, they are based on fundamentally different perspectives, despite of some overlap. Devictor et al. [16] mentioned the mismatching role of taxonomic and functional diversity to ecosystem estimation; that is, functional underestimation and taxonomic overestimation in ecoregions. Therefore, while taxonomic diversity indices cannot be completely replaced by functional diversity indices [85], they can be used to examine ecosystems. 

Although there were few statistically significant linear relationships between the functional diversity indices and environmental variables in this study (Figure 9), there has been a lot of research on this topic in recent years, and some studies have found that functional diversity is partially affected by the environment. For example, functional richness declines with disturbances such as urbanization [86]. The functional diversity index fluctuates in response to changes in the habitat environment in addition to disturbance, having a higher impact than spatial vectors [87]. FRic is affected by water temperature, substratum, precipitation, and water quality parameters such as pH and total nitrogen [87,88]; FEve is also affected by substratum [88]. Furthermore, partial functional diversity such as FEve is occasionally independent of disturbances such as land-use changes [89]. Functional diversity did not exhibit a linear relationship with changes in the environment in this study; however, it was expected to feature other forms of relation, such as unimodal.

### 4.4. Limitations of the Study

We revealed the relationships between taxonomic and functional diversities and environmental variables in reservoirs. However, we are aware of the limitations of this study which are commonly observed in studies using datasets obtained from a public database or literature. Our dataset was from the SEEL. In this monitoring program, benthic macroinvertebrates were collected with a dredge net by drawing the sediment on the bottom of reservoirs and to prevent loss of active macroinvertebrates [28,90]. Kick net sampling may be more efficient at sampling benthic macroinvertebrates than other methods, but kick net sampling is only feasible in shallow water [90]. Sampling with a dredge is typically used to characterize benthic macroinvertebrate assemblages in lakes [91]. de Mendoza and Catalan [92] determined the sampling points of lakes according to the habitat type, and integrated samples in each lake for further analyses, supporting the sampling strategy of our study. Thus, our sampling of benthic macroinvertebrates in the reservoirs with a dredge is justifiable [28,93]. Despite these potential limitations, our results are comparable between taxonomic and functional diversities and environmental variables in the study reservoirs. 

## 5. Conclusions

We discovered the relationships between taxonomic and functional diversity and environmental variables in reservoirs. Communities were classified into three clusters based on taxonomic diversity, reflecting differences in environmental conditions as well as taxonomic and functional diversity. Functional traits in the four categories were classified into three groups by the SOM learning process, demonstrating a connection, similarities, or a trade-off relationship between functional traits such as the number of generations per year, adult size, and lifespan. Network association analysis revealed relationships between functional traits, such as a medium lifespan with one generation per year. Our findings support the characteristics of species’ survival strategies such as r- and K-selection. Among the functional diversity indices, FRic had the strongest relationship with taxonomic diversity. When environmental variables were considered, a linear relationship between both taxonomic diversity and functional diversity indices such as taxa richness, abundance and FRic and environmental conditions was discovered. Taxonomic and functional diversity showed both comparable and different trends, indicating that both could be used to complement ecosystem analysis. From this perspective, our findings should prove useful in developing new criteria for assessing freshwater ecosystem health, as well as in evaluating and predicting future alteration of benthic macroinvertebrate communities facing anthropogenic disturbances.

## Figures and Tables

**Table 1:** Description of biological traits and trait modalities (modified from Poff et al. [31]).

Trait	Trait State (Modality)	Code	Abbreviation
Voltinism	Semivoltine (<1 generation/y)	Volt1	V1
	Univoltine (1 generation/y)	Volt2	V2
	Bi- or multivoltine (>1 generation/y)	Volt3	V3
Adult life span	Very short (<1 wk)	Life1	A1
	Short (<1 mo)	Life2	A2
	Long (>1 mo)	Life3	A3
Adult size	Small (<9 mm)	Size1	S1
	Medium (9–16 mm)	Size2	S2
	Large (>16 mm)	Size3	S3
Tropic habit	Filterer-collector	FFG1	F1
	Gatherer-collector	FFG2	F2
	Herbivore (scraper, piercer, and shedder)	FFG3	F3
	Predator (piercer and engulfer)	FFG4	F4
	Shredder (detritivore)	FFG5	F5

**Table 2:** Association rules for functional traits in Figure 7a. Abbreviations of traits were described in Table 1.

No.	Antecedent	Consequent	Lift	Support (%)
1	F3	S3	1.2	10.0
2	V1	S3	1.4	17.5
3	V1	A3	2.3	15.0

## Data Availability

The data are available on request from the corresponding author.

## Supplementary Materials

The following supporting information can be downloaded at: https://www.mdpi.com/article/10.3390/ijerph20010673/s1, Table S1: List of 20 study reservoirs in the Nakdong River catchment, South Korea. Table S2: List of taxa collected from 20 reservoirs in the Nakdong River catchment. Table S3: A list of references used for functional traits. References [30,31,32,33,34,35,94,95,96,97,98,99,100,101] are cited in the Supplementary Materials.

## References

[1] Simpson E.H. (1949). Measurement of diversity. Nature.

[2] Shannon C.E. (1948). A mathematical theory of communication. Bell Syst. Tech. J..

[3] Peet R.K. (1974). The measurement of species diversity. Annu. Rev. Ecol. Syst..

[4] Bae M.-J., Li F., Kwon Y.-S., Chung N., Choi H., Hwang S.-J., Park Y.-S. (2014). Concordance of diatom, macroinvertebrate and fish assemblages in streams at nested spatial scales: Implications for ecological integrity. Ecol. Indic..

[5] Menezes S., Baird D.J., Soares A.M.V.M. (2010). Beyond taxonomy: A review of macroinvertebrate trait-based community descriptors as tools for freshwater biomonitoring. J. Appl. Ecol..

[6] Tilman D., Levin S.A. (2001). Functional diversity. Encyclopedia of Biodiversity.

[7] Mason N.W.H., Mouillot D., Lee W.G., Wilson J.B. (2005). Functional richness, functional evenness and functional divergence: The primary components of functional diversity. Oikos.

[8] Villéger S., Mason N.W.H., Mouillot D. (2008). New multidimensional functional diversity indices for a multifaceted framework in functional ecology. Ecology.

[9] Moon M.Y., Ji C.W., Lee D.-S., Lee D.-Y., Hwang S.-J., Noh S.-Y., Kwak I.-S., Park Y.-S. (2020). Characterizing responses of biological trait and functional diversity of benthic macroinvertebrates to environmental variables to develop aquatic ecosystem health assessment index. Korean J. Ecol. Environ..

[10] Hooper D.U., Solan M., Symstad A., Díaz S., Gessener M.O., Buchmann N., Degrange V., Grime P., Hulot F., Mermillod-Blondin F., Loreau M., Naeem S., Inchausti P. (2002). Species diversity, functional diversity and ecosystem functioning. Biodiversity and Ecosystem Functioning: Synthesis and Perspectives.

[11] Cummins K.W. (1973). Trophic relations of aquatic insects. Annu. Rev. Entomol..

[12] Violle C., Navas M.-L., Vile D., Kazakou E., Fortunel C., Hummel I., Garnier E. (2007). Let the concept of trait be functional!. Oikos.

[13] Díaz S., Purvis A., Cornelissen J.H.C., Mace G.M., Donoghue M.J., Ewers R.M., Jordano P., Pearse W.D. (2013). Functional traits, the phylogeny of function, and ecosystem service vulnerability. Ecol. Evol..

[14] Tomanova S., Moya N., Oberdorff T. (2008). Using macroinvertebrate biological traits for assessing biotic integrity of neotropical streams. River Res. Appl..

[15] Villéger S., Miranda J.R., Hernández D.F., Mouillot D. (2010). Contrasting changes in taxonomic vs. functional diversity of tropical fish communities after habitat degradation. Ecol. Appl..

[16] Devictor V., Mouillot D., Meynard C., Jiguet F., Thuiller W., Mouquet N. (2010). Spatial mismatch and congruence between taxonomic, phylogenetic and functional diversity: The need for integrative conservation strategies in a changing world. Ecol. Lett..

[17] Jarzyna M.A., Jetz W. (2018). Taxonomic and functional diversity change is scale dependent. Nat. Commun..

[18] Bae M.-J., Kwon Y., Hwang S.-J., Chon T.-S., Yang H.-J., Kwak I.-S., Park J.-H., Ham S.-A., Park Y.-S. (2011). Relationships between three major stream assemblages and their environmental factors in multiple spatial scales. Ann. De Limnol.-Int. J. Limnol..

[19] Muralidharan M., Selvakumar C., Sudar S., Raja M. (2010). Macroinvertebrates as potential indicators of environmental quality. Int. J. Biol. Technol..

[20] Bae M.-J., Park Y.-S. (2016). Responses of the functional diversity of benthic macroinvertebrates to floods and droughts in small streams with different flow permanence. Inland Waters.

[21] Bae M.-J., Park Y.-S. (2019). Evaluation of precipitation impacts on benthic macroinvertebrate communities at three different stream types. Ecol. Indic..

[22] Biswas S.R., Mallik A.U. (2011). Species diversity and functional diversity relationship varies with disturbance intensity. Ecosphere.

[23] Fu X., Yang W., Zheng L., Liu D., Li X. (2022). Spatial patterns of macrobenthos taxonomic and functional diversity throughout the ecotones from river to lake: A case study in Northern China. Front. Ecol. Evol..

[24] Coccia C., Almeida B.A., Green A.J., Gutiérrez A.B., Carbonell J.A. (2021). Functional diversity of macroinvertebrates as a tool to evaluate wetland restoration. J. Appl. Ecol..

[25] Minister of Environment (MOE), National Institute of Environmental Research (NIER) (2017). Survey on the Environment and Ecosystem of Lakes.

[26] Bae M.-J., Park Y.-S. (2017). Diversity and distribution of endemic stream insects on a nationwide scale, South Korea: Conservation perspectives. Water.

[27] Lee D.-Y., Lee D.-S., Hwang S.-J., Lee K.-L., Park Y.-S. (2022). Distribution patterns and vulnerability of stoneflies (Plecoptera: Insecta) in South Korean streams with conservation perspectives. Glob. Ecol. Conserv..

[28] Kim S.-J., Song H.-J., Park T.-J., Hwang M.-Y., Cho H.-S., Song K.-D., Lee H.-J., Kim Y.-S. (2015). Survey on lake environments in the Yeongsan and Seomjin river basins-Based on 10 lakes such as Hadong and Sangsa. J. Korean Soc. Water Environ..

[29] QGIS (2022). org. QGIS Geographic Information Systemp.

[30] Phillips N. (2004). Stream biomonitoring using species traits. Water Atmos..

[31] Poff N.L., Olden J.D., Vieira N.K., Finn D.S., Simmons M.P., Kondratieff B.C. (2006). Functional trait niches of North American lotic insects: Traits-based ecological applications in light of phylogenetic relationships. J. N. Am. Benthol. Soc..

[32] Sarremejane R., Cid N., Stubbington R., Datry T., Alp M., Cañedo-Argüelles M., Cordero-Rivera A., Csabai Z., Gutiérrez-Cánovas C., Heino J. (2020). DISPERSE, a trait database to assess the dispersal potential of European aquatic macroinvertebrates. Sci. Data.

[33] Kwon S.J., Jun Y.-C., Park J.-H. (2013). Benthic Macroinvertebrates.

[34] U.S. EPA (2012). Freshwater Biological Traits Database (Final Report).

[35] Barbour M.T., Gerritsen J., Snyder B.D., Stribling J.B. (1999). Rapid Bioassessmnet Protocols for Use in Streams and Wadeable Rivers: Periphyton, Benthic Macroinvertebrates and Fish, Second Edition.

[36] Botsford L.W., Holland M.D., Samhouri J.F., White J.W., Hastings A. (2011). Importance of age structure in models of the response of upper trophic levels to fishing and climate change. ICES J. Mar. Sci..

[37] Öckinger E., Schweiger O., Crist T.O., Debinski D.M., Krauss J., Kuussaari M., Petersen J.D., Pöyry J., Settele J., Summerville K.S. (2010). Life-history traits predict species responses to habitat area and isolation: A cross-continental synthesis. Ecol. Lett..

[38] García-Barros E. (2008). Body size, egg size, and their interspecific relationships with ecological and life history traits in butterflies (Lepidoptera: Papilionoidea, Hesperioidea). Biol. J. Linn. Soc..

[39] Swanson S.K., Bahr J.M., Schwar M.T., Potter K.W. (2001). Two-way cluster analysis of geochemical data to constrain spring source waters. Chem. Geol..

[40] Tukey J.W. (1949). Comparing individual means in the analysis of variance. Biometrics.

[41] McCune B., Grace J., Urban D.L. (2002). Analysis of Ecological Communities.

[42] Kohonen T. (1982). Self-organized formation of topologically correct feature maps. Biol. Cybern..

[43] Vesanto J. (2000). Neural Network Tool for Data Mining: SOM toolbox.

[44] Céréghino R., Park Y.S. (2009). Review of the Self-Organizing Map (SOM) approach in water resources: Commentary. Environ. Model. Softw..

[45] Lee D.-Y., Lee D.-S., Bae M.-J., Hwang S.-J., Noh S.-Y., Moon J.-S., Park Y.-S. (2018). Distribution patterns of odonate assemblages in relation to environmental variables in streams of South Korea. Insects.

[46] Hahsler M., Grün B., Hornik K. (2005). arules-A computational environment for mining association rules and frequent item sets. J. Stat. Softw..

[47] Brin S., Motwani R., Ullman J.D., Tsur S. (1997). Dynamic itemset counting and implication rules for market basket data. Proceedings of the 1997 ACM SIGMOD International Conference on Management of Data.

[48] Hastie T.J., Tibshirani R.J. (1990). Generalized Additive Models.

[49] Laliberte E., Legendre P. (2010). A distance-based framework for measuring functional diversity from multiple traits. Ecology.

[50] Laliberte E., Legendre P., Shipley B. (2014). FD: Measuring Functional Diversity from Multiple Traits, and Other Tools for Functional Ecology. https://cran.r-project.org/web/packages/FD/FD.pdf.

[51] Oksanen J., Blanchet F.G., Friendly M., Kindt R., Legendre P., McGlinn D., Minchin P.R., O’Hara R.B., Simpson G.L., Solymos P. (2019). Vegan: Community Ecology Package. https://cran.r-project.org/web/packages/vegan/vegan.pdf.

[52] Wehrens R., Kruisselbrink J. (2018). Kohonen: Supervised and Unsupervised Self-Organising Maps. https://cran.r-project.org/web/packages/kohonen/kohonen.pdf.

[53] R Core Team (2021). R: A Language and Environment for Statistical Computing.

[54] Pohlert T. (2021). PMCMRplus: Calculate Pairwise Multiple Comparisons of Mean Rank Sums Extended. https://cran.r-project.org/web/packages/PMCMRplus/PMCMRplus.pdf.

[55] Hahsler M., Buchta C., Gruen B., Hornik K. (2022). Arules: Mining Association Rules and Frequent Itemsets, 1.7–4. https://cran.r-project.org/web/packages/arules/arules.pdf.

[56] Wood S.N. (2017). Generalized Additive Models: An Introduction with R.

[57] Wood S. (2022). Mgcv: Mixed GAM Computation Vehicle with Automatic Smoothness Estimation. https://cran.r-project.org/web/packages/mgcv/mgcv.pdf.

[58] Pangle K.L., Malinich T.D., Bunnell D.B., DeVries D.R., Ludsin S.A. (2012). Context-dependent planktivory: Interacting effects of turbidity and predation risk on adaptive foraging. Ecosphere.

[59] Sweka J.A., Hartman K.J. (2001). Influence of turbidity on brook trout reactive distance and foraging success. Trans. Am. Fish. Soc..

[60] Zhang Y., Cheng L., Li K., Zhang L., Cai Y., Wang X., Heino J. (2019). Nutrient enrichment homogenizes taxonomic and functional diversity of benthic macroinvertebrate assemblages in shallow lakes. Limnol. Oceanogr..

[61] Yang Y., Yi Y., Zhou Y., Wang X., Zhang S., Yang Z. (2020). Spatio-temporal variations of benthic macroinvertebrates and the driving environmental variables in a shallow lake. Ecol. Indic..

[62] Rawson C.A., Lim R.P., Tremblay L.A., Warne M.S.J., Ying G.-G., Laginestra E., Chapman J.C. (2010). Benthic macroinvertebrate assemblages in remediated wetlands around Sydney, Australia. Ecotoxicology.

[63] Park Y.-S., Kwon Y.-S., Hwang S.-J., Park S. (2014). Characterizing effects of landscape and morphometric factors on water quality of reservoirs using a self-organizing map. Environ. Model. Softw..

[64] Kwon Y.-S., Bae M.J., Kim J.-S., Kim Y.-J., Kim B.-H., Park Y.S. (2014). Characterizing changes of water quality and relationships with environmental factors in the selected Korean reservoirs. Korean J. Ecol. Environ..

[65] Blocksom K.A., Kurtenbach J.P., Klemm D.J., Fulk F.A., Cormier S.M. (2002). Development and evaluation of the lake macroinvertebrate integrity index (LMII) for New Jersey lakes and reservoirs. Environ. Monit. Assess..

[66] Spieles D.J., Mitsch W.J. (2003). A model of macroinvertebrate trophic structure and oxygen demand in freshwater wetlands. Ecol. Model..

[67] Lougheed V.L., Crosbie B., Chow-Fraser P. (2001). Primary determinants of macrophyte community structure in 62 marshes across the Great Lakes basin: Latitude, land use, and water quality effects. Can. J. Fish. Aquat. Sci..

[68] Usseglio-Polatera P., Bournaud M., Richoux P., Tachet H. (2000). Biological and ecological traits of benthic freshwater macroinvertebrates: Relationships and definition of groups with similar traits. Freshw. Biol..

[69] Firmiano K.R., Castro D.M.P., Linares M.S., Callisto M. (2021). Functional responses of aquatic invertebrates to anthropogenic stressors in riparian zones of Neotropical savanna streams. Sci. Total Environ..

[70] Pianka E.R. (1970). On r- and K-Selection. Am. Nat..

[71] Zeuss D., Brunzel S., Brandl R. (2017). Environmental drivers of voltinism and body size in insect assemblages across Europe. Glob. Ecol. Biogeogr..

[72] Yang F., Kawabata E., Tufail M., Brown J.J., Takeda M. (2017). r/K-like trade-off and voltinism discreteness: The implication to allochronic speciation in the fall webworm, *Hyphantria cunea* complex (Arctiidae). Ecol. Evol..

[73] Keppeler F.W., Montaña C.G., Winemiller K.O. (2020). The relationship between trophic level and body size in fishes depends on functional traits. Ecol. Monogr..

[74] Wallace J.B., Webster J.R. (1996). The role of macroinvertebrates in stream ecosystem function. Annu. Rev. Entomol..

[75] Gergs A., Ratte H.T. (2009). Predicting functional response and size selectivity of juvenile *Notonecta maculata* foraging on *Daphnia magna*. Ecol. Model..

[76] Pawar S., Dell A.I., Van M.S. (2012). Dimensionality of consumer search space drives trophic interaction strengths. Nature.

[77] Anderson N.H., Cummins K.W. (1979). Influences of diet on the life histories of aquatic insects. J. Fish. Res. Board Can..

[78] Wissinger S.A. (1988). Life history and size structure of larval dragonfly populations. J. N. Am. Benthol. Soc..

[79] Teder T., Tammaru T., Esperk T. (2008). Dependence of phenotypic variance in body size on environmental quality. Am. Nat..

[80] García-Navas V., Sattler T., Schmid H., Ozgul A. (2021). High elevation bird communities in the Swiss Alps exhibit reduced fecundity and lifespan independently of phylogenetic effects. Biodivers. Conserv..

[81] Collins S.L., Avolio M.L., Gries C., Hallett L.M., Koerner S.E., La Pierre K.J., Rypel A.L., Sokol E.R., Fey S.B., Flynn D.F.B. (2018). Temporal heterogeneity increases with spatial heterogeneity in ecological communities. Ecology.

[82] Lytle D.A. (2001). Disturbance regimes and life-history evolution. Am. Nat..

[83] Reynaga M.C., Dos Santos D.A. (2013). Contrasting taxonomical and functional responses of stream invertebrates across space and time in a Neotropical basin. Fundam. Appl. Limnol..

[84] Hacala A., Lafage D., Prinzing A., Sawtschuk J., Pétillon J. (2021). Drivers of taxonomic, functional and phylogenetic diversities in dominant ground-dwelling arthropods of coastal heathlands. Oecologia.

[85] Schmera D., Heino J., Podani J. (2022). Characterising functional strategies and trait space of freshwater macroinvertebrates. Sci. Rep..

[86] Barnum T.R., Weller D.E., Williams M. (2017). Urbanization reduces and homogenizes trait diversity in stream macroinvertebrate communities. Ecol. Appl..

[87] Li Z., Jiang X., Wang J., Meng X., Heino J., Xie Z. (2019). Multiple facets of stream macroinvertebrate alpha diversity are driven by different ecological factors across an extensive altitudinal gradient. Ecol. Evol..

[88] Heino J. (2005). Functional biodiversity of macroinvertebrate assemblages along major ecological gradients of boreal headwater streams. Freshw. Biol..

[89] Luck G.W., Carter A., Smallbone L. (2013). Changes in bird functional diversity across multiple land uses: Interpretations of functional redundancy depend on functional group identity. PLoS ONE.

[90] Klemm D.J., Lewis P.A., Fulk F., Lazorchak J.M. (1990). Macroinvertebrate Field and Laboratory Methods for Evaluating the Biological Integrity of Surface Waters.

[91] Hails J.R. (1982). Grap Samplers.

[92] de Mendoza G., Catalan J. (2010). Lake macroinvertebrates and the altitudinal environmental gradient in the Pyrenees. Hydrobiologia.

[93] Moore I.E., Murphy K.J. (2015). Evaluation of alternative macroinvertebrate sampling techniques for use in a new tropical freshwater bioassessment scheme. Acta Limnol. Bras..

[94] Li Z., Heino J., Liu Z., Meng X., Chen X., Ge Y., Xie Z. (2021). The drivers of multiple dimensions of stream macroinvertebrate beta diversity across a large montane landscape. Limnol. Oceanogr..

[95] Vieira N.M.K., Poff N.L., Carlisle D., Moulton Ii S.R., Koski M.L., Kondratieff B.C. (2006). A Database of Lotic Invertebrate Traits for North America.

[96] Tachet H., Richoux P., Bournaud M., Usseglio-Polatera P. (2000). Invertébrés d’eau Douce Systématique, Biologie, Écologie.

[97] Rodhain F. (1996). Ecology of Aedes aegypti in Africa and Asia. Bull. De La Soc. De Pathol. Exot..

[98] Koch M. (2004). Observations of the reproduction and population structure of the caenogastropod, *Gabbia vertiginosa* Frauenfeld, 1862 (Rissooidea: Bithyniidae). Molluscan Res..

[99] Villalobos M.C., Monge-Nájera J., Barrientos Z., Franco J. (2015). Life cycle and field abundance of the snail *Succinea costaricana* (Stylommatophora: Succineidae), a tropical agricultural pest. Rev. De Biol. Trop..

[100] Kuznik-Kowalska E., Pokryszko B.M., Prockow M., Oczkowska M. (2013). On the population dynamics, reproductive biology and growth of *Succinea putris* (Linnaeus 1758)(Gastropoda: Pulmonata: Succineidae). Folia Malacol..

[101] Jeliazkov A. (2013). Scale-Effects in Agriculture-Environment-Biodiversity Relationships.

