# Supplementary material for: Taxonomic and Functional Diversity of Benthic Macroinvertebrate Assemblages in Reservoirs of South Korea
Source: Int J Environ Res Public Health. 2022 Dec 30;20(1):673. doi: 10.3390/ijerph20010673 (PMC9819676; doi:10.3390/ijerph20010673)
Supplement: Supplementary file 1 [file ijerph-20-00673-s001.zip › ijerph-1984327-supplementary.pdf]

Table S1. List of taxa collected from 20 reservoirs in the Nakdong River catchment, South Korea.

Reservoir	Acronym	Location (GPS)	Elevation (m)	Sampled Year (No. sampling)
Andong	AnDn	36°7'48.51"N,128°50'12.18"E	155	2016(2)
Angye	AnGy	36°0'35.60"N,129°15'50.71"E	45	2016(2)
Bomun	BoMn	35°50'39.72"N,129°16'32.19"E	97	2009(2)
Bongsan	BnSn	35°26'2.93"N,128°29'28.77"E	8	2014(1);2017(1)
Daeam	DaAm	35°32'25.83"N,129°10'43.91"E	51	2016(2)
Deokdong	DkDn	35°50'15.86"N,129°18'40.38"E	167	2009(2)
Gucheon	GuCh	34°49'31.14"N,128°38'14.02"E	92	2009(2)
Gyeongcheon	GyCh	36°42'20.90"N,128°18'39.01"E	233	2014(2)
Hapcheon	HpCh	35°33'55.08"N,128°2'13.70"E	181	2009(2)
Hoeya	HoYa	35°28'15.87"N,129°16'11.64"E	30	2016(2)
Imha	ImHa	36°31'32.53"N,128°53'28.02"E	158	2009(2)
Jangcheok	JnCh	35°25'55.02"N,128°29'39.25"E	13	2014(1),2017(1)
Jinyang	JnYa	35°9'25.45"N,127°59'12.91"E	39	2017(2)
Otae	OoTa	36°29'29.13"N,128°7'17.49"E	82	2009(2)
Sayeon	SaYn	35°35'14.25"N,129°11'9.11"E	58	2016(2)
Seonam	SnAm	35°30'58.55"N,129°19'43.54"E	25	2016(2)
Unmun	WnMn	35°43'50.57"N,128°56'13.01"E	134	2017(2)
Yeoncho	YnCh	34°56'13.31"N,128°40'16.67"E	44	2009(2)
Yeongcheon	YnCn	36°4'16.09"N,129°1'36.09"E	153	2017(2)
Yongyeon	YoYn	36°07'38.01"N,129°17'45.06"E	57	2016(2)

Table S2. List of taxa collected from 20 reservoirs in the Nakdong River catchment, South Korea.

Class	Order	Taxa	Code
Insecta	Coleoptera	*Laccophilus difficilis*	C01
Insecta	Coleoptera	*Hydaticus grammicus*	C02
Insecta	Coleoptera	*Berosus lewisius*	C03
Insecta	Coleoptera	Haliplidae spp.	C04
Insecta	Coleoptera	Helodidae spp.	C05
Insecta	Coleoptera	*Amphiops mater*	C06
Insecta	Coleoptera	*Rhantus pulverosus*	C07
Insecta	Coleoptera	*Enochrus simulans*	C08
Insecta	Coleoptera	*Sternolophus rufipes*	C09
Insecta	Coleoptera	Elmidae spp.	C10
Insecta	Coleoptera	*Galerucella nipponensis*	C11
Insecta	Coleoptera	*Noterus japonicus*	C12
Insecta	Coleoptera	*Laccobius* spp.	C13
Insecta	Coleoptera	*Helochares striatus*	C14
Insecta	Coleoptera	*Peltodytes sinensis*	C15
Insecta	Diptera	*Tipula* sp. 1	D01
Insecta	Diptera	*Chironomus* spp.	D02
Insecta	Diptera	Stratiomyiidae spp.	D03
Insecta	Diptera	Ceratopogonidae spp.	D04
Insecta	Diptera	*Anopheles* spp.	D05
Insecta	Diptera	Ephydridae spp.	D06
Insecta	Diptera	Dixidae spp.	D07
Insecta	Diptera	*Aedes* spp.	D08
Insecta	Diptera	*Anopheles* spp.	D09
Insecta	Diptera	Dolichopodidae spp.	D10
Insecta	Diptera	*Stratiomys japonica*	D11
Insecta	Diptera	*Culicini* spp.	D12
Insecta	Diptera	*Chaoborius* spp.	D13
Insecta	Ephemeroptera	*Procloeon pennulatum*	E01
Insecta	Ephemeroptera	*Baetis fuscatus*	E02
Insecta	Ephemeroptera	*Ephemera orientalis*	E03
Insecta	Ephemeroptera	*Caenis* sp. 1	E04
Insecta	Ephemeroptera	*Cloeon dipterum*	E05
Insecta	Ephemeroptera	*Potamanthus formosus*	E06
Insecta	Ephemeroptera	*Ecdyonurus dracon*	E07
Insecta	Hemiptera	*Diplonychus esakii*	H01
Insecta	Hemiptera	*Ranatra chinensis*	H02
Insecta	Hemiptera	*Plea indistinguenda*	H03
Insecta	Hemiptera	*Micronecta sedula*	H04
Insecta	Hemiptera	*Micronecta* spp.	H05
Insecta	Hemiptera	*Muljarus japonicus*	H06
Insecta	Hemiptera	*Sigara substriata*	H07
Insecta	Hemiptera	*Aquarius paludum*	H08
Insecta	Lepidoptera	*Cataclysta* spp.	L01
Insecta	Odonata	*Trigomphus nigripes*	O01
Insecta	Odonata	*Nihonogomphus* sp. 1	O02
Insecta	Odonata	*Crocothemis servilia*	O03
Insecta	Odonata	*Ceriagrion melanurum*	O04

Insecta	Odonata	*Macromia daimoji*	O05
Insecta	Odonata	*Pseudothemis zonata*	O06
Insecta	Odonata	*Paracercion calamorum*	O07
Insecta	Odonata	*Paracercion hieroglyphicum*	O08
Insecta	Odonata	*Orthetrum albistylum*	O09
Insecta	Odonata	*Deielia phaon*	O10
Insecta	Odonata	*Platycnemis phillopoda*	O11
Insecta	Odonata	*Epophthalmia elegans*	O12
Insecta	Odonata	Coenagrionidae spp.	O13
Insecta	Odonata	*Ischnura asiatica*	O14
Insecta	Odonata	*Anax parthenope julius*	O15
Insecta	Trichoptera	*Cheumatopsyche brevilineata*	T01
Insecta	Trichoptera	*Molanna moesta*	T02
Insecta	Trichoptera	*Ecnomus tenellus*	T03
Insecta	Trichoptera	*Mystacides* sp. 1	T04
Bivalvia	Unionoida	*Unio douglasiae*	N01
Bivalvia	Veneroida	*Corbicula fluminea*	N02
Clitellata	Arhynchobdellida	*Erpobdella lineata*	N03
Clitellata	Arhynchobdellida	*Hirudo nipponia*	N04
Clitellata	Rhynchobdellida	*Glossiphonia complanata*	N05
Clitellata	Rhynchobdellida	*Alboglossiphonia lata*	N06
Clitellata	Tubificida	*Limnodrilus* spp.	N07
Clitellata	Tubificida	*Branchiura sowerbyi*	N08
Gastropoda	Architaenioglossa	*Sinotaia quadrata*	N09
Gastropoda	Architaenioglossa	*Cipangopaludina chinensis*	N10
Gastropoda	Architaenioglossa	*Pomacea canaliculata*	N11
Gastropoda	Littorinimorpha	*Parafossarulus manchouricus*	N12
Gastropoda	Systellommatophora	*Gyraulus chinensis*	N13
Gastropoda	Systellommatophora	*Radix auricularia*	N14
Gastropoda	Systellommatophora	*Laevapex nipponica*	N15
Gastropoda	Systellommatophora	*Polypylis hemisphaerula*	N16
Gastropoda	Systellommatophora	*Hippeutis cantori*	N17
Gastropoda	Systellommatophora	*Austropeplea ollula*	N18
Gastropoda	Systellommatophora	*Physa acuta*	N19
Gastropoda	Unassigned	*Semisulcospira gottschei*	N20
Gastropoda	Unassigned	*Semisulcospira libertina*	N21
Gastropoda	Unassigned	*Semisulcospira forticosta*	N22
Malacostraca	Decapoda	*Caridina denticulata*	N23
Malacostraca	Decapoda	*Palaemon paucidens*	N24
Malacostraca	Decapoda	*Macrobrachium nipponense*	N25
Malacostraca	Isopoda	Asellidae spp.	N26
Rhynchobdellida	Rhynchobdellida	*Torix tagoi*	N27

Table S3. A list of references used for functional traits. References are given in References in the main text.

Trait	Reference
Voltinism	[30], [31], [32], [94], [95], [96], [97], [98], [99], [100], [101]
Adult life span	[30], [31], [32], [94], [95], [98], [99], [100]
Size at maturity	[30], [31], [32], [97]
Functional feeding groups	[30], [31], [33], [34], [35]
